# Genomic organization and evolution of the Atlantic salmon hemoglobin repertoire

**DOI:** 10.1186/1471-2164-11-539

**Published:** 2010-10-05

**Authors:** Nicole L Quinn, Keith A Boroevich, Krzysztof P Lubieniecki, William Chow, Evelyn A Davidson, Ruth B Phillips, Ben F Koop, William S Davidson

**Affiliations:** 1 Department of Molecular Biology and Biochemistry, Simon Fraser University, Burnaby, British Columbia, Canada; 2 Department of Biological Sciences, Washington State University, Vancouver, WA, USA; 3 Department of Biology, University of Victoria, Victoria, British Columbia, Canada

## Abstract

**Background:**

The genomes of salmonids are considered pseudo-tetraploid undergoing reversion to a stable diploid state. Given the genome duplication and extensive biological data available for salmonids, they are excellent model organisms for studying comparative genomics, evolutionary processes, fates of duplicated genes and the genetic and physiological processes associated with complex behavioral phenotypes. The evolution of the tetrapod hemoglobin genes is well studied; however, little is known about the genomic organization and evolution of teleost hemoglobin genes, particularly those of salmonids. The Atlantic salmon serves as a representative salmonid species for genomics studies. Given the well documented role of hemoglobin in adaptation to varied environmental conditions as well as its use as a model protein for evolutionary analyses, an understanding of the genomic structure and organization of the Atlantic salmon α and β hemoglobin genes is of great interest.

**Results:**

We identified four bacterial artificial chromosomes (BACs) comprising two hemoglobin gene clusters spanning the entire α and β hemoglobin gene repertoire of the Atlantic salmon genome. Their chromosomal locations were established using fluorescence *in situ *hybridization (FISH) analysis and linkage mapping, demonstrating that the two clusters are located on separate chromosomes. The BACs were sequenced and assembled into scaffolds, which were annotated for putatively functional and pseudogenized hemoglobin-like genes. This revealed that the tail-to-tail organization and alternating pattern of the α and β hemoglobin genes are well conserved in both clusters, as well as that the Atlantic salmon genome houses substantially more hemoglobin genes, including non-Bohr β globin genes, than the genomes of other teleosts that have been sequenced.

**Conclusions:**

We suggest that the most parsimonious evolutionary path leading to the present organization of the Atlantic salmon hemoglobin genes involves the loss of a single hemoglobin gene cluster after the whole genome duplication (WGD) at the base of the teleost radiation but prior to the salmonid-specific WGD, which then produced the duplicated copies seen today. We also propose that the relatively high number of hemoglobin genes as well as the presence of non-Bohr β hemoglobin genes may be due to the dynamic life history of salmon and the diverse environmental conditions that the species encounters.

Data deposition: BACs S0155C07 and S0079J05 (fps135): GenBank GQ898924; BACs S0055H05 and S0014B03 (fps1046): GenBank GQ898925

## Background

Hemoglobin, one of the most well-studied proteins to date, is responsible for oxygen transport from the lungs or gills to the tissues of vertebrates. The hemoglobin molecule is comprised of two α and two β subunits that non-covalently bond to form a tetramer [1,2]. The genes encoding the hemoglobin subunits are abundantly present and show relatively high similarity in structure (i.e., consisting of three exons and two introns) throughout the vertebrate lineage [3]. These characteristics, combined with the relative ease of isolating and studying hemoglobin proteins and their suspected role in adaptation to variable environmental conditions, have made the hemoglobin genes major targets for evolutionary studies [1-4].

Examinations of the genomic organization of chromosomal hemoglobin gene regions suggest that all hemoglobin genes evolved from a single monomeric form when gnathostome fish evolved from the more primitive agnathan fish approximately 500-700 million years ago [5,6]. The entire hemoglobin gene region then appears to have undergone a series of tandem duplications and divergence, giving rise to the modern α and β hemoglobin genes. Initially the α and β genes were adjacent on the same chromosome, and expansion of this region, including lineage-specific gene gain and loss, produced the multiple copies of α and β genes seen in Gnathostomata today [4,5,7,8].

The current genomic organization seen in mammals and birds is such that α and β hemoglobin gene clusters are located on different chromosomes and transcribed from the same strand in order of temporal expression [8,9]. The most parsimonious explanation of this arrangement involves a disruption in the α-β linkage by translocation of part of the hemoglobin gene cluster and subsequent gene silencing of α and β hemoglobins on respective chromosomes prior to the lineage leading to birds and mammals approximately 300-350 million years ago [9,10]. Studies of the genomic organization of hemoglobin genes in the mammalian and avian lines examined this hypothesis by looking for evolutionary "footprints" of silenced hemoglobin genes as well as conservation and divergence patterns of genes surrounding the α and β gene clusters along the mammalian line [9,11].

The disruption in the α and β hemoglobin gene linkage in mammals and birds appears to have occurred after their divergence from the poikilothermic jawed vertebrate taxa. Rather, with the exception of some extreme cold-adapted Antarctic icefish that retain only remnants of α hemoglobin genes and have completely lost the β hemoglobin genes, and thus do not express hemoglobin [12,13], the fish and amphibians studied to date exhibit intermixed α and β hemoglobin genes on the same chromosome. For example, within the amphibian line, the genomes of both *Xenopus laevis *and *X. tropicalis *exhibit linked α and β hemoglobin genes [10].

The teleosts, or the ray-finned fish, are a diverse group that comprises most living species of fish, including more than 20,000 extant species covering more than 40 orders [14]. Despite significant differences in the number of hemoglobin genes and within-chromosome arrangements, model teleosts whose genomes have been studied to date, including the Japanese pufferfish (*Fugu rubripes*) [15], the zebrafish (*Danio rerio*) [16] and medaka (*Orzias latipes*) [17] are reported to exhibit two hemoglobin gene clusters located on distinct chromosomes. These observations support the hypothesis that the teleost lineage experienced a whole genome duplication (WGD) event subsequent to the divergence from tetrapods [18].

The Salmonidae, a family of teleosts that includes the salmon, trout, charr, grayling and whitefish, are of considerable environmental, economic and social importance. Indeed, more is known about the biology of salmonids than any other fish group [19]. The common ancestor of salmonids underwent a WGD event between 20 and 120 million years ago [20,21]. Thus, the extant salmonid species are considered pseudo-tetraploids whose genomes are in the process of reverting to a stable diploid state. The Atlantic salmon (*Salmo salar*) has been chosen as a representative salmonid for genomics studies, and an international collaboration to sequence the Atlantic salmon genome has been established [22].

Wolff and Gannon [23] provided the first sequence of an Atlantic salmon α hemoglobin from a kidney cDNA library. Subsequently, reports of the organization of the Atlantic salmon hemoglobin gene cluster described six lambda phage genomic clones comprising two sets of α and β hemoglobin genes oriented 3' to 3' on opposite strands [24-26]. This was the first evidence of this type of hemoglobin gene arrangement for any vertebrate species. The six clones comprised four unique α hemoglobin gene sequences and six unique β hemoglobin genes, including a β hemoglobin containing the characteristic amino acid changes that eliminate the Bohr effect, as well as one partial β hemoglobin gene (GenBank accession numbers X97284-X97289) [26]. It remained unknown, however, whether these represented all hemoglobin-like genes within the Atlantic salmon genome. In addition, it was not known whether the clusters were on separate chromosomes, or whether, as would be predicted by the salmonid-specific 4R WGD hypothesis [27], there were actually four hemoglobin gene clusters in salmon. Furthermore, the relative locations and orders of the clones to one another were not established, and the sequences of intergenic regions as well as the genes surrounding the hemoglobins were not determined. Finally, these investigations were not able to identify putative pseudogenes, incomplete hemoglobin genes or footprints of historical hemoglobin genes within the hemoglobin gene clusters or elsewhere in the genome. Thus, a full characterization of the Atlantic salmon hemoglobin gene repertoire is needed to provide insight to the evolution of the organization and function of the Atlantic salmon hemoglobins, particularly in light of the teleost and salmonid-specific WGD events.

We used oligonucleotide probes specific for Atlantic salmon α, β and non-Bohr β hemoglobin genes as well as probes designed from rainbow trout (*Oncorhynchus mykiss*) embryonic hemoglobin cDNAs [28] to locate these genes within the Atlantic salmon bacterial artificial chromosome (BAC) library, CHORI-214 [29]. Four BACs, representing two genomic locations on different chromosomes and comprising the entire Altantic salmon hemoglobin gene repertoire were sequenced and annotated. Fluorescence *in situ *hybridization (FISH) and linkage analyses were performed to assign these BACs to chromosomal locations within the Atlantic salmon genome. Here we present the first description of an entire salmonid α and β hemoglobin gene repertoire. We also discuss our results in terms of the fate of the hemoglobin genes during and after the salmonid WGD event, and how this fits into the evolution of the hemoglobin gene family in teleosts.

## Results

### Identification and tiling paths of Atlantic salmon hemoglobin-containing BACs

All ^32^P-labelled 40-mer probes for α, β, non-Bohr β and embryonic hemoglobins (probe and primer sequences are provided in Additional file 1, Table S1) hybridized to Atlantic salmon BACs belonging to two fingerprint scaffolds (fps), within the Atlantic salmon physical map [30,31]. Fps1046 contains 21 BACs and spans an estimated 458.8 kb; fps135 is comprised of 391 BACs spanning approximately 3.473 Mb. PCR was used to confirm the hybridization results and narrow down the regions within the fps that contained hemoglobin genes by screening all BACs surrounding the hybridization-positive BACs for the presence of hemoglobin genes. PCR primers were designed for sequence tag sites (STS) within the BAC-end sequences (SP6 and T7 ends) of suspected overlapping BACs spanning the hemoglobin gene region, and overlaps were checked by PCR amplification of the STS within the putative overlapping BACs. The overlapping BACs S0014B03 and S0055H05 were determined to span the hemoglobin gene region of fps1046, while S0155C07 and S0079J05 spanned that of fps135, thus creating BAC tiling paths for the hemoglobin regions of these fps. Individual shotgun libraries were generated for all four BACs and sequenced.

### Sequence assemblies and annotation

**Figure 1:**
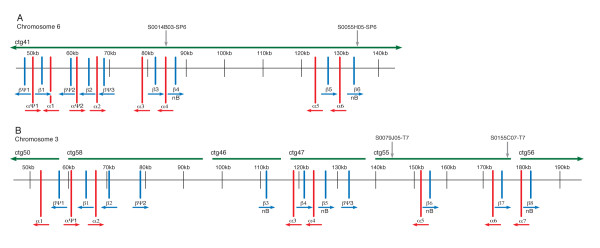
**Genomic organization of the Atlantic salmon hemoglobin gene clusters**. A) Schematic representation of the region of Atlantic salmon chromosome 6 containing the hemoglobin genes. Sequence reads for this region assembled into one solid sequence contig (ctg 41). B) Schematic representation of the region of Atlantic salmon chromosome 3 containing the hemoglobin genes. Sequence contigs are indicated by horizontal green lines. β hemoglobin genes are indicated in blue; α hemoglobin genes are indicated in red. Arrows indicate strand of transcription. All hemoglobin gene names begin with SsaChr6 or SsaChr3 for chromosome 6 and chromosome 3, respectively, followed by α or β and a number indicating the order of the genes. SP6 and T7 ends of overlapping BACs are indicated by grey arrows. Thus, the regions between the arrows indicate BAC overlapping regions. bN: Non-Bohr β hemoglobins.

The CHORI-214 BAC library was made from a diploid male Atlantic salmon individual, meaning that BAC inserts originated from either maternal or paternal chromosomes and therefore, overlapping BACs could exhibit allelic differences. This appeared to be the case for BACs S0014B03 and S0055H05, for which the overlapping region covered the hemoglobin genes within fps1046. Thus, although this overlapping section assembled into one contiguous section, reads containing allelic differences assembled into independent contigs that aligned to homologous regions along the solid contig. Nevertheless, the full BACs assembled very well, and only three contigs > 1000 bp and two gaps remained after hand finishing. These gaps presumably span repetitive regions in the Atlantic salmon genome [32]. Furthermore, given that the entire hemoglobin gene region was assembled with no gaps, it can be assumed that the six putatively functional α and six putatively functional β hemoglobin genes, two of which were defined as non-Bohr β hemoglobins, along with the two putative α hemoglobin pseudogenes and three putative β hemoglobin pseudogenes that were annotated within fps1046 represent all hemoglobin genes within that cluster (note that the solid contig was used for sequence annotation; Figure 1A). The total size of the assembly for the two BACs, not including allelic contigs (i.e., the non-redundant sequence), was 242,883 bp, with approximately 49,000 bp of overlap between them and the hemoglobin genes spanning approximately 87,000 bp.

Sequence reads from the overlapping region between the BACs S0155C07 and S0079J05 of fps135 assembled into one sequence contig with no apparent allelic differences. However, the remainder of the fps135 BACs proved much more difficult to assemble, and unfortunately, the repetitive nature of the sequences made it impossible to further improve the assembly by sequencing PCR products to fill gaps because we were unable to design specific PCR primers that would amplify a single product (i.e., numerous bands, or smears on agarose gels were obtained). Thus, a total of 23 sequence contigs > 1000 bp remained after hand-finishing of the assembly. The relative orders of some of the sequence contigs of fps135 were determined by matching paired-ends of sequence shotgun clones. Six sequence contigs contained hemoglobin genes, and the relative order of these fps135 contigs with respect to one another was estimated by aligning the contigs against the completed assembly of the two BACs from fps1046. This was based on the assumption, given the highly similar nature of the non-coding regions, as well as that of the genes flanking the globins, that there has not been a major disruption in the form of an inversion to either of the hemoglobin regions (i.e., that of fps135 or fps1046). Our sequence annotation identified seven putatively functional α hemoglobin genes and one putative α hemoglobin pseudogene, as well as eight putatively functional β hemoglobin genes, four of which were defined as non-Bohr β hemoglobins, and three putative β hemoglobin pseudogenes within the fps135 hemoglobin BACs (Figure 1B). This, however, must be considered a minimum estimate of hemoglobin genes within this region given the possibility that gaps between sequence contigs could contain additional hemoglobin genes. The total size of the assembled sequence contigs for the two BACs was 421,907 bp, with approximately 33,000 bp of overlap. The hemoglobin genes spanned approximately 130,000 bp not including gaps between contigs.

All sequences were deposited in the NCBI GenBank database with the assembled sequence contigs for BACs S0155C07 and S0079J05 (fps135) under the accession number GQ898924 and those for BACs S0055H05 and S0014B03 (fps1046) under the accession number GQ898925.

All previously published Atlantic salmon hemoglobin sequences [25] were identified within the annotated hemoglobin clusters; however, there were two examples of possible allelic differences. Specifically, Clone 3 α hemoglobin (GenBank accession number X97286.1) exhibited 99% similarity at the nucleotide level to SsaChr6α6, which resulted in one amino acid change from a methionine to a leucine at amino acid 32, and the Clone 5 and Clone 6 β hemoglobins (Bohr; X97288 and X97289) showed 99% similarity at the nucleotide level with SsaChr6β3, which resulted in one amino acid change from valine to leucine at amino acid 143. However, as both of these changes were caused by single nucleotide substitutions, each resulting in a single amino acid change, and given the depth of sequencing coverage of the Atlantic salmon BACs (i.e., > 18× coverage in both cases), it is more probable that they reflect sequencing errors in the published clones rather than allelic differences.

To provide further evidence that the two Atlantic salmon hemoglobin gene clusters encompassed all previously identified Atlantic salmon α and β hemoglobin genes, we compared all identified putatively functional Atlantic salmon hemoglobin genes against all full-length Atlantic salmon cDNA clones [33]. Indeed, all unique full-length cDNA clones annotated as β hemoglobins were accounted for within the identified putatively functional β hemoglobin genes. This was also true for the α hemoglobins, with the exception of the cDNA clones with accession numbers BT046755.1 and BT046550.1, which are highly similar to one another but not to the identified hemoglobins. An alignment of these clones using BLASTn [34] against the nr/nt database revealed similarity to hemoglobin subunit α-D, a distinct type of hemoglobin present in birds, mammals and reptiles that is predicted to have arisen via duplication from a gene that had larval/embryonic function [35]. This gene is apparently found in Atlantic salmon given the presence of the ESTs, but is not in the regions of the α and β hemoglobin genes.

Additional file 2, Table S2 lists all annotated α hemoglobin genes (Table S2A) and β hemoglobin genes (Table S2B) with the source chromosome, strand of transcription, start location, whether the entire hemoglobin gene matches one of the Atlantic salmon hemoglobin clones published by McMorrow et al. [25] at the amino acid level. It also identifies which genes are non-Bohr β hemoglobins, and lists the top hemoglobin EST cluster hit, if any, with the percent identity from the salmonid EST database [33,36] and whether the hemoglobin matches one of the full-length cDNA clones with the corresponding NCBI accession number. Table S2C (Additional file 2) lists all putative pseudogenes with the chromosome name, strand of transcription, start location and a description of each exon, with explanations of why the gene was classified as a pseudogene.

The identified putatively functional α hemoglobin genes SsaChr6α2 SsaChr3α2 as well as the β genes SsaChr6β1, SsaChr6β2, SsaChr6β3, SsaChr6β6, SsaChr3β1, SsaChr3β2 and SsaChr3β8 did not have matching EST clones. This could mean that these represent newly identified hemoglobin genes, or that these genes are rarely or never transcribed and thus are not represented in the Atlantic salmon cDNA libraries. Interestingly, several of these genes lie in regions where the tail-to-tail alternating order of the hemoglobin genes is disrupted and they would be transcribed on opposite strands than expected (see below for more details). Future studies using expression profiling of the Atlantic salmon transcriptome at various time points throughout the species' life cycle will provide further insight to this.

### Conservation of gene order and strand of transcription

Wagner et al. [24] first reported the tail-to-tail orientation and alternating order of the Atlantic salmon α and β hemoglobin genes. We found that this orientation was fairly well conserved, with the α hemoglobins transcribed on the negative strand and β hemoglobins transcribed on the positive strand, and the alternating α-β order was mostly maintained in both chromosomes with some notable exceptions. On chromosome 6 (fps1046), the alternating α-β pattern is conserved throughout (including putative pseudogenes), but there are some apparent disruptions to the strand of transcription at the 5' end of the cluster. Specifically, SsaChr6β2 as well as all putative β hemoglobin pseudogenes, including SsaChr6βψ1, SsaChr6βψ2, SsaChr6βψ3, were predicted as being transcribed from the negative strand, whereas all putative α hemoglobin pseudogenes (SsaChr6αψ1, SsaChr6αψ2) as well as SsaChr6α2 would be transcribed on the positive strand (Figure 1A). On chromosome 3 (fps135), the β hemoglobin pseudogene SsaChr3βψ1 as well as the putatively functional genes SsaChr3β1 and SsaChr3β2 were predicted to be transcribed from the negative strand, and SsaChr3βψ2 and SsaChr3βψ3 disrupt the otherwise conserved alternating α-β order and orientation of the genes, SsaChr3β2 and SsaChr3β3 being adjacent (Figure 1B). In terms of α hemoglobin genes on chromosome 3, SsaChr3αψ1 and SsaChr3α2 were predicted as transcribed in the positive direction, whereas all others are transcribed on the negative strand. Note again that, for chromosome 3, the order and orientation of the sequence contigs was predicted based on homology with that of chromosome 6 (see dot plot in Additional file 3, Figure S1). Thus, it is possible that inversions or rearrangements may have taken place, and that the resulting predicted order and orientation of the hemoglobin genes is incorrect.

As indicated above, it is interesting to note that all of the putatively functional α hemoglobin genes predicted to be transcribed from the positive strand and all β hemoglobin genes predicted to be transcribed from the negative strand are lacking a corresponding EST at this time. It is possible that the apparent rearrangements have contributed to a global shutdown of transcription in these regions of the genome, allowing several of the hemoglobin genes to degenerate into obvious pseudogenes and silencing the remainder. This should be further explored using expression profiling by qPCR across all life stages of Atlantic salmon.

### Linkage analysis and karyotyping

**Figure 2:**
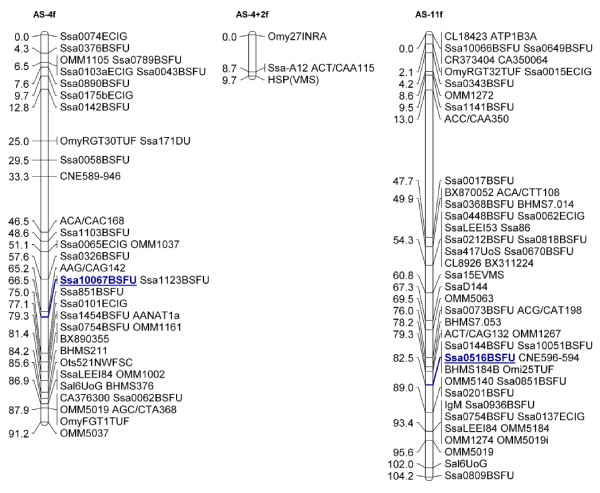
**Merged female linkage maps for Atlantic salmon SALMAP families Br5 and Br6 showing linkage groups 4 and 11**. Microsatellite marker Ssa10067BSFU (underlined), representing fps1046 was informative in both the Altantic salmon SALMAP families (Br5 and Br6) and mapped to linkage group 4. Microsatellite Ssa0516BSFU (underlined) was informative in the Br6 family and mapped to linkage group 11.

Microsatellite marker Ssa10067BSFU, representing fps1046 was informative in both the Atlantic salmon SALMAP families (Br5 and Br6) [37,38] and was mapped to linkage group 4. Microsatellite Ssa0516BSFU was informative in the Br6 family and mapped to linkage group 11 (Figure 2). FISH analysis revealed that fps1046 is found within Atlantic chromosome 6 and fps135 is within chromosome 3 (see [38] for chromosome nomenclature). The FISH and linkage mapping of chromosomes 6 and 3 to linkage groups 4 and 11, respectively, contributed to the integration of the Atlantic salmon karyotype and linkage map [38]. Primer sequences for the microsatellite markers used for linkage analysis are provided in Additional File 1, Table S1 and within ASalbase, the Atlantic salmon genomic database [31].

### Comparative genomic analysis of hemoglobin gene regions in other teleosts

**Figure 3:**
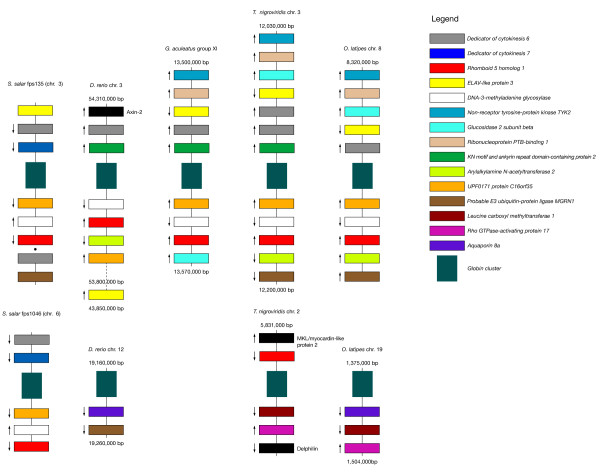
**Comparative synteny of hemoglobin gene clusters among sequenced teleost species**. Schematic representation of annotated genes within the regions surrounding the hemoglobin gene clusters for Atlantic salmon and four annotated teleost genomes (*O. latipes, D. rerio, G. aculeatus, T. nigroviridis*). Colored blocks indicate shared or common genes as specified in the Figure legend. Black blocks indicate genes that are not shared within the indicated regions of any other species. Distances between genes vary (i.e., figure is not to scale); the start and end of the chromsome/group region is shown in base pairs (bp) for each of the annotated teleost genomes. Solid lines between predicted genes indicate that the order and orientation of the predicted gene relative to those neighboring it is known, whereas for Atlantic salmon fps 135 (chromosome 3), a single black dot between predicted genes indicates that the relative location of the predicted genes compared to those joined by solid lines is known, but their order and orientation (i.e., that of the sequence contigs on which they reside) relative to one another is not. Arrows indicate the direction of transcription of the gene relative to the location of the hemoglobin gene cluster; lack of an arrow indicates that the relative direction of transcription cannot be determined. For *D. rerio *chromsome 3, the gene for *ELAV-like protein *was found distantly downstream of the nearest common gene (*Arylakylamine N-acetyltransferase 2*), as indicated by the distance shown, with numerous predicted genes in between.

We examined the regions surrounding the hemoglobin gene clusters in the available teleost genomes and compared them against one another as well as to those predicted to surround the Atlantic salmon hemoglobin gene clusters to gain insight to the nature of the teleostean hemoglobin gene containing chromosomes. We found that the genes surrounding the hemoglobin gene clusters are well conserved. Figure 3 shows a schematic diagram of the hemoglobin gene regions and the predicted surrounding named genes in medaka, zebrafish, stickleback and tetraodon compared to those of Atlantic salmon. Note that, for the hemoglobin gene containing BACs within Atlantic salmon chromosome 3, only the order and orientation of the sequence contigs that aligned with those from the BACs within chromosome 6 could be predicted. That is, given the extensive overlap between the two BACs that cover fps1046 (chromosome 6), the total sequenced region is much shorter than that of fps135 (chromosome 3); therefore, any sequence contigs from fps135 that did not fall within the coverage of fps1046 could not be ordered or oriented. Thus, for any sequence contigs that fell outside of this region, we were only able to establish their relative location compared to those that aligned with chromosome 3 based on their source BAC. Within Figure 3, solid lines between predicted genes indicate that the order and orientation of the predicted gene relative to those neighboring it is known, whereas a single black dot between predicted genes indicates that the relative location of the predicted genes compared to those joined by solid lines is known, but their order and orientation (i.e., that of the sequence contigs on which they reside) relative to one another is not. Arrows in Figure 3 indicate the direction of transcription of the gene relative to the location of the hemoglobin gene cluster; lack of an arrow indicates that the relative direction of transcription cannot be determined.

Briefly, medaka, zebrafish and tetraodon and Atlantic salmon exhibit two distinct hemoglobin gene clusters on separate chromosomes or linkage groups, whereas stickleback has only one. Although there are some rearrangements in terms of the positioning of genes relative to the hemoglobin genes and direction of transcription as well as some apparent gains, losses and duplications of genes, all of the organisms possess one similar cluster (hereafter Cluster 1) that contains, among others, the shared genes *UPF0171 protein C16orf35*, *Rhomboid family member 1*, *Dedicator of cytokinesis protein 6*, *ELAV-like protein 3 *and *DNA-3-methyladenine glycosylase *(*MPG*; see Figure 3). Note these results are consistent with those of Patel et al. [11], who report that *MPG *and *C16orf35 *surround the α hemoglobin gene cluster in frog, chicken and human, and one of the α and β hemoglobin clusters in platypus and opposum. However, whereas this cluster appears twice in Atlantic salmon, the second cluster in zebrafish, tetraodon and medaka (hereafter Cluster 2) is characterized by a different set of shared genes; specifically, the presence of *Aquaporin-8 *and *Rho-GTPase-activating protein*, although tetraodon is lacking the former and zebrafish is lacking the latter. In addition, tetraodon exhibits a copy of *Rhomboid family member 1 *on Cluster 2 as well as Cluster 1. Stickleback and Atlantic salmon, however, appear to have lost Cluster 2 entirely. Instead, the stickleback genome only has one hemoglobin cluster (Cluster 1), whereas that of Altantic salmon shows two copies of Cluster 1.

A dot plot generated using the JDotter software [39] comparing the sequenced BACs from Atlantic salmon chromosomes 3 and 6 showed that the regions surrounding the hemoglobin genes are > 95% similar between the two chromosomes, with variations only within the hemoglobin gene regions (Additional file 3, Figure S1). This further suggests that the two Atlantic salmon hemoglobin gene containing chromosomes or regions are homeologous (i.e., represent duplicated copies of the same cluster as the result of a WGD event). Thus, we hypothesize that the WGD at the base of the teleost lineage produced Cluster 1 and Cluster 2, which remain in the zebrafish, medaka and tetraodon lineages, that Cluster 2 was lost in the stickleback lineage, and that Cluster 2 was lost within the salmonid lineage prior to the WGD, which yielded two copies of Cluster 1. This hypothesis is also supported by the fact that the Atlantic salmon chromosome arms 3q and 6q (where the hemoglobin gene clusters are located) share nine duplicated genetic markers [38].

### Phylogenetic analysis of teleostean hemoglobin genes

**Figure 4:**
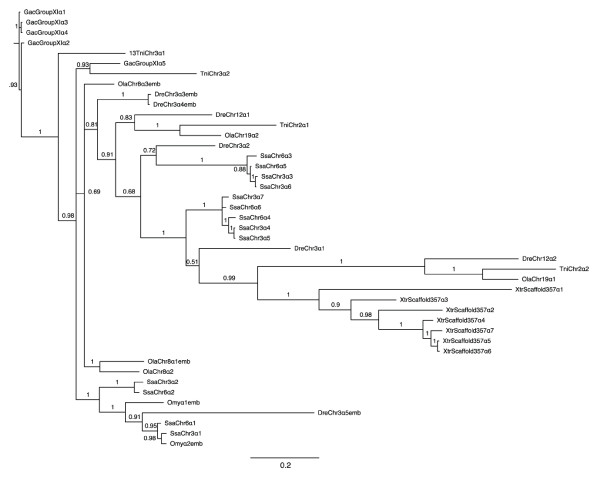
**Phylogenetic tree of teleost and *Xenopus tropicalis *α hemoglobins**. The α hemoglobin cDNAs (exclusive of untranslated regions) annotated within the Ensembl 54 database for medaka, zebrafish, tetraodon, stickleback and *X. tropicalis*, as well as those identified in Atlantic salmon here and the hemoglobin genes identified as embryonic within rainbow trout [28] were independently aligned using EBioX [70]. Phylogenetic trees were constructed using the a Bayesian approach with (5 runs, 100,000 generations, 40% burn-in period) within the TOPALi V.2 software package [71] running the MrBayes program [72] under the best selected model (SYM). For simplicity, as well as to clearly indicate the source chromosome of the gene, the teleostean hemoglobin genes were named using the same system used to name those of Atlantic salmon. That is, an abbreviated three letter (genus species) name followed by chromosome/linkage group name followed by α or β followed by a number indicating the sequential order of the genes from 5' to 3' as defined by Ensembl (Additional file 4, Table S3). Hemoglobin genes that were previously identified via expression analysis as being expressed exclusively during embryogenesis, and that are identified as embryonic within the Ensembl 54 database are denoted with "emb" following the assigned gene name. Branch numbers indicate posterior probabilities.

**Figure 5:**
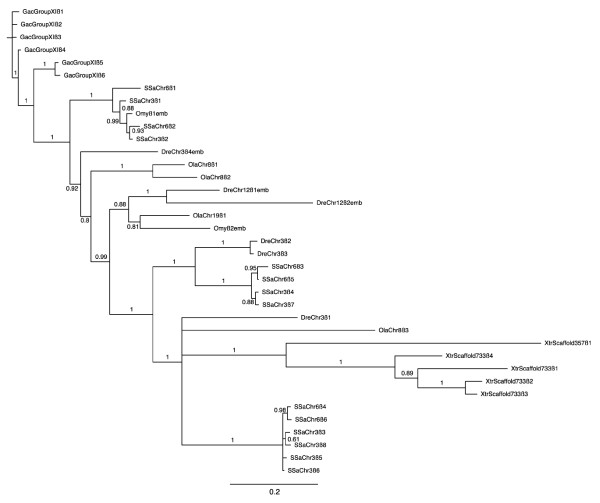
**Phylogenetic tree of teleost and *Xenopus tropicalis *β hemoglobins**. The β hemoglobin cDNAs (exclusive of untranslated regions) annotated within the Ensembl 54 database for medaka, zebrafish, tetraodon, stickleback and *X. tropicalis*, as well as those identified in Atlantic salmon here and the hemoglobin genes identified as embryonic within rainbow trout [28] were independently aligned using EBioX [70]. Phylogenetic trees were constructed using the a Bayesian approach with (5 runs, 100,000 generations, 40% burn-in period) within the TOPALi V.2 software package [71] running the MrBayes program [72] under the best selected model (SYM). For simplicity, as well as to clearly indicate the source chromosome of the gene, the teleostean hemoglobin genes were named using the same system used to name those of Atlantic salmon. That is, an abbreviated three letter (genus species) name followed by chromosome/linkage group name followed by α or β followed by a number indicating the sequential order of the genes from 5' to 3' as defined by Ensembl (Additional file 4, Table S3). Hemoglobin genes that were previously identified via expression analysis as being expressed exclusively during embryogenesis, and that are identified as embryonic within the Ensembl 54 database are denoted with "emb" following the assigned gene name. Branch numbers indicate posterior probabilities.

The results of the phylogentic analysis (Figures 4 and 5 for α and β genes, respectively) suggest that the hemoglobin genes cluster according to functional similarity, which corresponds to sequence similarity. This is expected given the high sequence similarity and short nature of the hemoglobin genes. Specifically, in Figure 5, all of the non-Bohr α hemoglobin genes (SsaChr3β3, SsaChr3β5, SsaChr3β6, SsaChr3β8, SsaChr6β4 and SsaChr6β6) form a distinct clade with no other hemoglobin genes, further supporting that there are no β globin genes lacking the Bohr effect in the other fish species examined (see Discussion). Additionally, many genes that were annotated as embryonic within Ensembl (identified with "emb" following the species name) clustered closely, which provides some suggestion as to candidate Atlantic salmon embryonic hemoglobin genes (see Discussion).

In both trees, the *X. tropicalis *hemoglobin genes formed their own clade, although they did not form distinct outgroups, which again, may be a function of the high similarity between the hemoglobin genes across species. All annotated medaka, zebrafish, stickleback and tetraodon α and β hemoglobin genes that were used to generate the phylogenetic trees (i.e., all that were identified within the Ensembl 54 database) are provided in Additional file 4, Table S3 by species with the corresponding name assigned by us for comparison purposes (see Methods), as well as the Ensembl gene ID, chromosome/linkage group, start and stop location and strand of transcription.

## Discussion

### Number of hemoglobin gene clusters and whole genome duplications

**Figure 6:**
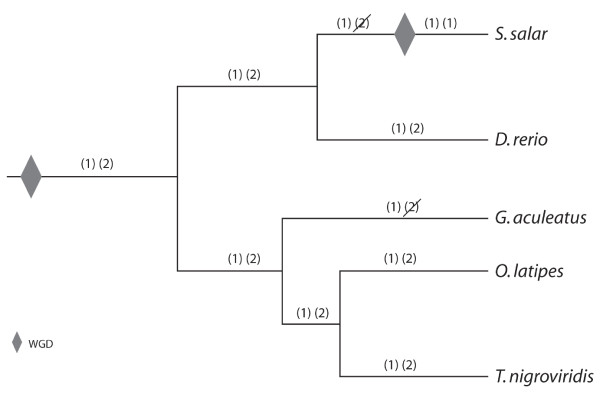
**Schematic representation of the evolution of teleostean hemoglobin gene clusters**. Whole genome duplication (WGD) events are indicated by grey diamonds. The two hemoglobin gene clusters resulting from the teleost WGD are represented as (1) and (2) for Cluster 1 and Cluster 2, respectively (see text). Loss of a hemoglobin gene cluster by excision is indicated by a diagonal slash across that cluster. Although the genome sequence is available for the pufferfish, *T. rubripes*, the fugu genome was not included in this analysis because the published hemoglobin arrangement of two hemoglobin gene clusters, one containing only α hemoglobin genes and one containing both α and β hemoglobin genes [15] did not agree with the annotation results of the latest fugu genome assembly reported within the Ensembl database.

Notably, there are not four clusters of hemoglobin genes in Atlantic salmon even though the hemoglobin clusters were already duplicated within the teleost lineage prior to the salmonid-specific WGD event that took place between 20 and 120 million years ago [20,21]. Furthermore, our comparative genomic analysis, as well as the high similarity in the non-coding regions of the two fps, suggests that Atlantic salmon exhibit a duplicated copy of one cluster (Cluster 1), and are missing the second cluster (Cluster 2), which is still seen in medaka, zebrafish and tetraodon (see Figures 3 and 6). Figure 3 shows a schematic diagram of the predicted genes surrounding the hemoglobin genes in Atlantic salmon as well as four annotated teleost genomes, while Figure 6 depicts the phylogenetic relationships of the studied teleost fishes (adapted from [40]), and illustrates our hypothesis of the evolutionary events that took place to produce the observed chromosomal arrangements of the teleost hemoglobin genes. With respect to the other teleosts studied, subsequent to the teleost WGD, which produced Clusters 1 and 2, zebrafish and medaka and tetraodon appear to have maintained both hemoglobin gene clusters, whereas stickleback and Atlantic salmon have lost Cluster 2. In addition, zebrafish exhibits an apparent inversion in Cluster 1 such that *ELAV-like protein 3 *is located on the opposite side of the hemoglobin genes, far downstream from *Rhomboid family member 1 *with several unshared genes between them. Within the tetraodon genome, Cluster 2 also exhibits some shuffling compared to those of the other genomes, and, interestingly, contains *Rhomboid family member 1*, which is also found on Cluster 1. These relationships will be clarified by further analysis and in-depth annotation of the full-length hemoglobin gene repertoires of other teleost species as more of them undergo full genome sequencing.

With respect to the salmonid lineage, we propose that Atlantic salmon lost Cluster 2 prior to the salmonid-specific WGD, which duplicated Cluster 1, thus producing the two copies of Cluster 1 and lack of Cluster 2 seen within the Atlantic salmon genome today. We also recognize the possibility of an alternative pathway, which involves tetraploidization of hemoglobin gene Clusters 1 and 2 as predicted by the salmonid-specific WGD (i.e., producing two copies of each cluster), followed by subsequent loss of both copies of Cluster 2 within the salmonid lineage. However, this involves two separate excision events subsequent to the salmonid WGD, whereas the former hypothesis only involves one such event prior to the salmonid WGD, and is thus the more parsimonious route. Note that the loss of the two clusters must have taken place by excision of the entire regions or chromosome loss as opposed to gradual degradation of the hemoglobin genes or gene silencing, or we would have expected our hemoglobin probes to hybridize to footprints of old hemoglobin clusters in other regions of the genome.

### Conservation of order and orientation of α and β hemoglobin genes

The tail-to-tail orientation and alternating order of the α and β hemoglobin genes were fairly well conserved throughout both hemoglobin gene clusters, although both hemoglobin gene clusters exhibited some apparent disruptions to these patterns (see Results). However, given that no hemoglobin gene footprints or ghost genes could be found within these regions, we predict that these changes took place via gradual shuffling, gene loss via excision and pseudogenization over time. Such lineage-specific gains and loses of hemoglobin genes have also been reported throughout the mammalian lineage [8,35]

### Number of hemoglobin genes in Atlantic salmon

Our results revealed that the Atlantic salmon genome contains substantially more paralogous α and β hemoglobin gene copies than previously published [24-26]. Furthermore, there are more copies of the α and β hemoglobin genes within the Atlantic salmon genome than in other teleosts whose genomes have been sequenced. Specifically, of the teleost genomes examined, that of zebrafish contains the most hemoglobin genes, with six β hemoglobin genes and seven α hemoglobin genes, compared to 13 and 14 putatively functional α and β hemoglobin genes, respectively, in Atlantic salmon. Note, however, that some of the putatively functional hemoglobin genes do not have an EST associated with them, perhaps suggesting that the actual number of functional genes should be reduced accordingly. However, this would still leave the Atlantic salmon with more α and β hemoglobin genes than any of the other teleosts examined to date.

Numerous reports suggest that mammalian hemoglobin levels are implicated in the increased oxygen affinity of blood in situations of adaptation to altitude-induced hypoxia (reviewed by [41]), thus suggesting that hemoglobin levels contribute to survival in low oxygen environments. Further, Hoffman et al. [35] suggest that variation in hemoglobin gene copy number may be a source of regulatory variation affecting physiological differences in blood oxygen transport and aerobic energy metabolism in mammals. Indeed, it has been proposed that the capacity of fish to colonize a wide range of habitats is directly related to their hemoglobin systems [42]. In addition, a study using real-time quantitative PCR demonstrated that hypoxic conditions induce complex responses in hemoglobin gene expression in zebrafish [43]. Thus, the extensive array of hemoglobins in Atlantic salmon may reflect the diverse range of environmental conditions that an individual salmon must endure throughout its lifecycle as it migrates from freshwater streams to open ocean and back.

### Identification of β hemoglobins lacking the Bohr effect

The Bohr effect is the phenomenon that the affinity of hemoglobin for O_2 _is affected by pH. Specifically, an increase in the blood pCO_2 _shifts the oxygen dissociation curve to the right, resulting in the release of O_2 _and thereby enabling more efficient gas exchange between blood and tissues. This is the result of the oxy-deoxy conformational change and allosteric interactions between O_2 _and H^+^/CO_2 _binding sites of the hemoglobin molecule. Hemoglobin molecules lacking the Bohr effect are able to retain O_2 _under conditions of elevated acidity caused by increased oxygen consumption [44]. Therefore, the non-Bohr hemoglobin may function as an emergency oxygen supplier when an organism is exercising vigorously, such as when a fish is escaping a predator, catching prey, or swimming against a current.

The Bohr effect depends on the intricate arrangement and interactions of all cation and anion binding sites in the hemoglobin molecule and involves a number of contributing amino acid groups that have not yet been fully elucidated [45]. Indeed, a comparison of mammalian, avian and teleost fish hemoglobins suggested that several different histidine and non-histidine sites contribute to the Bohr effect in different species to varying degrees [45]. It is widely accepted, however, that a greater overall histidine content in the hemoglobin molecule correlates with an increased Bohr effect [46], with the C-terminal histidine residue accounting for up to 50% of the effect [45,47,48]. In Atlantic salmon, the non-Bohr β hemoglobin exhibits phenylalanine at this position [25]. In addition, in Atlantic salmon, the non-Bohr β hemoglobin has 147 amino acids (vs. 148 in the Bohr molecule), including the initiator methoinine, and the amino acid at position 93 is alanine [26,49]. We used these three characteristics to identify a total of six putatively functional non-Bohr β hemoglobin genes within the Atlantic salmon genome (Figure 1; Additional file 2, Table S2B). Conversely, no β hemoglobin genes identified within the medaka, zebrafish, stickleback or tetraodon genomes exhibited all three of these hallmarks of the non-Bohr β hemoglobin. In addition, a recent PCR-based exploration of the Atlantic cod genome found no β hemoglobin genes exhibiting these characteristics [50], implying an absence of the non-Bohr hemoglobins in this species. All of the Atlantic salmon non-Bohr β hemoglobins formed a distinct clade in the phylogenetic analysis, which reflects their common structural elements and therefore highly similar sequences, and lends further support to the finding that no other fish species examined possesses non-Bohr hemoglobin genes [Figure 5].

This apparently high number of non-Bohr β hemoglobin gene copies within the Atlantic salmon genome may be attributable to the Atlantic salmon life history, with its extensive migratory range and the need to swim upstream into fresh water habitats to spawn. In contrast, all of the model teleosts studied so far inhabit relatively consistent environments with little variation in depth, temperature or salinity. Additionally, although Atlantic cod, a non-model teleost that does not appear to possess non-Bohr β hemoglobin genes, inhabit depths from the surface up to 600 m and undertake seasonal migration, at no point in their lifecycle do they inhabit freshwater [50]. Thus, future studies of the hemoglobin gene repertoires of other migratory salmonids, such as the Pacific salmon species, as well as land-locked freshwater salmonids, in addition to expression profiling of hemoglobin genes at different life stages will provide further insight to this phenomenon.

### Embryonic hemoglobin genes

To date, there has been no published study examining temporal expression of hemoglobin genes in Atlantic salmon. Indeed, the most closely related species for which this has been done is rainbow trout [28], for which there is no published genome sequence as well as no comprehensive examination of the rainbow trout hemoglobin repertoire such as this one. Given the complex life history of salmon and the lack of available expression data, we could not confidently assign the title of embryonic to any Atlantic salmon hemoglobins at this time. However, it is noteworthy that SsaChr3α1 and SsaChr6α1 form a clade with Omyα2emb, as well as Omyα1emb and an embryonic *Danio rerio *α globin, DreChr3α5emb (Figure 4), and that SsaChr3α2 and SsaChr6α2 cluster closely with this clade. Figure 5 shows that SsaChr3β1, SsaChr6β1, SsaChr3β2 and SsaChr6β2 form a clade with Omyβ1emb. These phylogenetic relationships suggest that these Atlantic salmon hemoglobin genes may be embryonic. Also worth noting is that all of these are the first genes in the 5'-3' direction on their respective chromosomes. In mammals, temporal expression of hemoglobin genes correlates with spatial location on the chromosome, with the first upstream hemoglobin gene being the first expressed [3]. This further suggests that these genes (i.e., SsaChr3α1 and SsaChr3α2, SsaChr6α1, SsaChr6α2, SsaChr3β1 SsaChr3β2, SsaChr6β1 and SsaChr6β2) encode candidate embryonic hemoglobins. However, further analysis, in particular, detailed expression profiling of hemoglobin genes during all life stages, is required to examine these hypotheses.

## Conclusions

We found that, despite the Atlantic salmon genome having gone through at least two WGD events relative to tetrapods, which would result in four predicted hemoglobin gene clusters, only two such clusters were present. Furthermore, the Atlantic salmon genome appears to exhibit two copies of one of the duplicated ancestral teleost hemoglobin gene clusters, and has presumably lost the other cluster. We also found that the Atlantic salmon genome harbors substantially more hemoglobin genes than the other teleosts for which the hemoglobin gene repertoires have been identified, and that they possess several hemoglobin genes that appear to encode non- Bohr β hemoglobins. We suggest that these characteristics of the Atlantic salmon hemoglobin genes reflect the dynamic life history of Atlantic salmon.

## Methods

### Identification of Atlantic salmon hemoglobin BACs

As part of the Genomic Research on All Salmonids Project (GRASP), an Atlantic salmon BAC library was produced from a partial EcoRI restriction enzyme digest of DNA from a Norwegian aquaculture strain male fish (CHORI-214 segments 1-3). There are 312,000 BAC clones in the library with an average insert size of 190,000 bp, which have been arrayed onto nylon membranes, thus representing an 18.8-fold coverage of the Atlantic salmon genome [29]. BACs were fingerprinted using HindIII and arranged into contigs to create the first physical map of a salmonid genome [30]. Approximately 210,000 BAC end-sequences have been determined, corresponding to approximately 3.5% of the Atlantic salmon genome. Information on the Atlantic salmon BACs and physical map can be found at [31].

To identify the Atlantic salmon BACs containing the hemoglobin genes, oligonucleotide probes (~40-mers) were designed from the published Atlantic salmon Clone 6 (GenBank accession number X97289) for α, β and non-Bohr β hemoglobins. PCR primers sets were also designed to span intron 1 of all three hemoglobin types. In addition, primer sets were designed to span intron 1 of the embryonic α and β hemoglobins of rainbow trout, with a ~40-mer forward primer that was used for hybridization probing (GenBank accession numbers: α: AB015448; β: AB015450). All primers and probes were designed using Primer3 ver. 0.4.0 [51] and are provided in Additional file 1, Table S1. The oligonucleotide probes were end-labeled with ^32^PγATP using T4 polynucleotide kinase and hybridized to six BAC filters at a time as described by Johnstone et al. [52]. Briefly, prehybridization was carried out in 5× saline-sodium citrate buffer (SSC), 0.5% sodium dodecyl sulfate (SDS), and 5 × Denhardt's solution at 65°C. The filters were washed three times for 1 hr at 50°C, in 1 × SSC and 0.1% SDS. Filters were exposed to phosphor screens that were scanned using the Typhoon Imaging System and visualized using ImageQuant software, giving an image of the ^32^P-labeled hybridization-positive BACs containing the hemoglobin markers. The hybridization-positive BAC clones were picked from the library, cultured in 5 mL LB media containing chloramphenicol (50 μg/mL) overnight at 37°C shaking at 250 rpm and made into glycerol stocks for subsequent PCR verification that they indeed contained hemoglobin genes. Hybridization and PCR-positive BACs for the hemoglobin genes were matched to two fingerprint scaffolds (fps) within the Altantic salmon physical map (fps135 and fps1046; [30,31]).

### BAC shotgun library generation and sequencing

Using a combination of hybridization probing and PCR (see above) to screen all BACs within the suspected hemoglobin gene containing regions, we identified two overlapping BACs from each of fps1046 (BACs S0055H05 and S0014B03) and fps135 (BACs S0155C07 and S0079J05) spanning the entire Atlantic salmon hemoglobin gene repertoire. That is, all primers amplified hemoglobin gene products within these BACs, and no additional BACs that were not contained within the four BACs as determined by the Atlantic salmon physical map yielded PCR products using the hemoglobin gene primers. The four BACs were sequenced using standard Sanger sequencing of a shotgun library as previously described [53]. Briefly, BAC DNA was isolated from each of the hemoglobin-containing BACs using Qiagen's Large Construct kit as per the manufacturer's directions (Qiagen, Mississauga, Ont. Canada). The kit includes an exonuclease digestion step to eliminate *E. coli *genomic DNA. The purified BAC DNA was sheared by sonication and blunt-end repaired. The sonicated DNA was size fractioned by agarose gel electrophoresis and 2-5 kb fragments were purified using the QIAquick Gel Extraction Kit (Qiagen, Mississauga, Ont. Canada). DNA fragments were ligated into pUC19 plasmid that had been digested with SmaI and treated with shrimp-alkaline phosphatase to produce de-phosphorylated blunt ends. The ligation mixture was used to transform supercompetent *E. coli *cells (XL1-Blue; Stratagene, La Jolla, CA. USA). Transformed cells were cultured overnight at 37°C on LB/agar plates supplemented with ampicillin (200 μg/mL) and 1,920 (5 × 384 well plates) clones were sent to the Michael Smith Genome Sciences Centre, Vancouver, BC Canada, for sequencing. The sequences were analyzed for quality using PHRED [54], assembled using PHRAP [55], and viewed using Consed version 15.0 [56]. BAC assemblies were complicated by the repetitive nature of the Atlantic salmon genome [32]. Assemblies were hand-finished to fill gaps (i.e., join sequence contigs) as best as possible using primer walking; however, primers could not be designed to join some sequence contigs that ended in repetitive sequence, or often primers amplified multiple products (i.e., showed a multiple bands or a smear on an agarose gel).

### Linkage analysis and chromosome assignment

The sequences of BACs S0055H05 and S0155C07 (representing fps 1046 and 135, respectively) were screened for microsatellite markers that were variable (i.e., informative) within the two Atlantic salmon SALMAP mapping families, Br5 and Br6, each of which contains two parents and 46 offspring [37]. Markers Ssa10067BSFU and Ssa10051BSFU were identified within S0055H05 and S0155C07, respectively. PCR primers were designed to amplify the region containing the microsatellite. The forward primer for each pair contained an M13 sequence tag that was used for genotyping analysis. Genotyping results were analyzed with LINKMFEX ver. 2.3 [57].

A single end-sequenced BAC containing the α, β and non-Bohr β hemoglobin genes was chosen from each of fps135 and fps1046 (S0155C07 and S0055H05, respectively) to be used for chromosome assignment. Approximately 1 μg of BAC DNA was purified (Qiagen mini-prep kit; Qiagen, Mississauga, Ont., Canada) and used for FISH analysis to identify the Atlantic salmon chromosomes containing the hemoglobins. Comparison of the results of the linkage and FISH analysis of the Atlantic salmon hemoglobin BACs contributed to the recent integration of the Atlantic salmon linkage map and karyotype [38].

### BAC sequence annotation and identification of putatively functional and pseudogenized hemoglobin genes

All sequence contigs > 1,000 bp within the assembled sequences were analyzed using a variety of sequence similarity searches and gene prediction algorithms that have been incorporated into an in-house computational pipeline and database [58] described previously [53]. Briefly, sequences entering this pipeline were screened (masked) for repetitive elements using RepeatMasker 3.2.6 [59] and were searched against the NCBI nr (non-redundant) and Atlantic salmon EST [33] databases using BLAST [34]. A GENSCAN gene model prediction algorithm [60] was used to predict introns and exons, and the resulting predictions were searched against the Uniref50 (clustered sets of sequences from UniProt Knowledgebase) database [61]. Finally, a rps-BLAST search against the NCBI CDD [62] was conducted to provide additional information with respect to the predicted genes. Any sequence contigs that were identified as containing hemoglobin-like genes by this pipeline were put through an additional series of annotation steps to ensure consistent calling of predicted open reading frames (ORFs) and that we did not miss any putatively functional or dysfunctional hemoglobin-like genes. Specifically, the masked and unmasked sequences were analysed using the ab initio gene prediction programs GENSCAN [60], GeneMark [63], FGENESH [64] and HMMGene [65], and the results of each prediction program were compared. In an attempt to identify putative psuedogenes or hemoglobin gene remnants, HMMer (v1.8.5) [66] was used to scan the sequence with hemoglobin exon-specific HMMs. Hemoglobin genes were labeled as putatively functional if they were predicted to be intact hemoglobins by our annotation procedures, and met all of the following criteria:

1) The genes were predicted to contain three exons and two introns.

2) The predicted exons were of the appropriate sizes, meaning that predicted splice junction sites aligned with those of known functional hemoglobins and start and stop codons were present in the appropriate places.

3) The final predicted protein included 147 or 148 amino acids for β hemoglobins and 143 amino acids for α hemoglobins.

The sequences of any predicted ORFs that aligned to hemoglobins but failed to meet any one of the above criteria were examined by eye for potential miss-calling by our annotation procedures. Specifically, we looked for historical footprints of missing exons that were not recognized by the pipeline, interruptions to splice sites as well as insertions and deletions of stop and start codons, potential sequencing errors and frame-shift mutations caused by insertions or deletions. We also examined by eye any putative three-exon ORFs identified by our pipeline that were not recognized by a BLAST search as encoding hemoglobins to determine whether they may be remnant hemoglobin genes or previously undefined hemoglobin-like genes. If, after this hands-on annotation, predicted proteins still did not meet the above criteria, the sequences were defined as putative pseudogenes. Furthermore, any regions for which the predicted orientation (i.e., α hemoglobin genes transcribed on the negative strand and β hemoglobin genes on the positive) and alternating order of the α and β hemoglobin genes was disrupted were examined by eye for putative remnant hemoglobin exons and introns. All such regions were aligned against intact hemoglobin genes using ClustalW2 [67], and predictions were made as to whether these regions represented footprints of historical hemoglobin genes.

All annotated hemoglobin genes were assigned an Ssa (*Salmo salar*) name followed by Chr3 for fps135 and Chr6 for fps1046 to denote its chromosomal location, then α or β to identify the gene encoded, and finally a number corresponding to its order relative to the other α or β genes on that chromosome from 5' to 3'.

### Identification of β hemoglobins lacking the Bohr effect

We defined β hemoglobin genes exhibiting three hallmarks of a lack of the Bohr effect were defined as putative non-Bohr β hemoglobin genes. These hallmarks include: 1) the non-Bohr β hemoglobin has 147 amino acids, including the initiator methionine; 2) the C-terminal amino acid is phenylalanine; 3) the amino acid at position 93 is alanine [48,27].

### Identification of genes surrounding hemoglobin gene clusters in Atlantic salmon and other teleosts

Atlantic salmon BAC sequences surrounding the hemoglobin gene clusters were annotated using our in-house annotation pipeline described above. This provided a preliminary prediction of the genes lying within the sequenced regions. However, note that different components of the pipeline can differ in their gene predictions, and that a full, comprehensive annotation of these regions as well as the rest of the Atlantic salmon genome will be completed with sequencing of the whole genome.

The genes surrounding the hemoglobin clusters in four annotated teleost genomes, medaka, zebrafish, tetraodon (*Tetraodon nigroviridis*) and stickleback (*Gasterosteus aculeatus*), were identified using the Pfam ID for the hemoglobin protein family (PF00042) [68] available within Biomart [69]. Specifically, the Ensembl 54 Genes database was searched using the appropriate genome-specific dataset for hemoglobins. Once the genomic locations of the hemoglobin genes were determined, the region surrounding the hemoglobin gene clusters was expanded until at least five predicted genes were identified on either side of the hemoglobin gene cluster, or until no additional common or shared genes could be identified. This allowed us to examine the synteny of the regions surrounding the hemoglobin genes, and thereby generate hypotheses of hemoglobin gene evolution in teleost fishes. Note that the fugu genome was not included in this analysis because the published hemoglobin arrangement of two hemoglobin gene clusters, one containing only α hemoglobin genes and one containing both α and β hemoglobins [15] did not agree with the annotation results of the latest fugu genome assembly reported within the Ensembl database. Instead, only one apparent hemoglobin cluster containing both α and β hemoglobin genes could be identified on fugu scaffold 3, and when the genes surrounding this cluster were compared to those of the other genomes examined, no shared genes (i.e., no conserved synteny) could be found.

### Phylogenetic analyses

The α and β hemoglobin cDNAs (exclusive of untranslated regions) annotated within the Ensembl 54 database for medaka, zebrafish, tetraodon and stickleback, as well as those identified in Atlantic salmon here and the hemoglobin genes identified as embryonic within rainbow trout [28] were independently aligned using EBioX [70]. We examined the relationships among the gene products by constructing phylogenetic trees using the a Bayesian approach with (5 runs, 100,000 generations, 40% burn-in period) within the TOPALi V.2 software package [71] running the MrBayes program [72] under the best selected model (SYM). For simplicity, as well as to clearly indicate the source chromosome of the gene, the teleostean hemoglobin genes were named using the same system used to name those of Atlantic salmon. That is, an abbreviated three letter (genus species) name followed by chromosome/linkage group name followed by α or β followed by a number indicating the sequential order of the genes from 5' to 3' as defined by Ensembl (Additional file 4, Table S3). Note that hemoglobin genes of medaka [73], zebrafish [16] and rainbow trout [28] that were previously identified via expression analysis as being expressed exclusively during embryogenesis, and that are identified as embryonic within the Ensembl 54 database are identified within the phylogenetic trees (denoted with "emb" following the assigned gene name) as well as within Additional file 4, Table S3.

## Authors' contributions

NLQ contributed to the study design, identification and isolation of BACs, sequence assembly and hand-finishing, sequence annotation, data analysis and manuscript preparation. KAB and WC conducted the bioinformatics and contributed to sequence annotation and data analysis. KPL assisted with sequence assembly and data analysis. EAD performed the linkage analysis and RBP conducted the FISH analysis. BFK and WSD contributed to the study design, data analysis and manuscript preparation. All authors read and approved the final manuscript.

## Supplementary Material

Additional file 1 Table S1: Primer and probe sequences. ^a ^~40-mer forward primers were also used as hybridization probes. Click here for file

Additional file 2 **Table S2A-C**: Identified Atlantic salmon putatively functional and pseudogenized hemoglobin genes. S2A) Identified putatively functional Atlantic salmon α hemoglobin genes with chromosome, sequence contig number and approximate location (kb), strand of transcription, most highly similar Atlantic salmon EST cluster (if any), whether the gene has a corresponding full-length EST, whether the gene matches any of the previously published Atlantic salmon hemoglobin genes at the amino acid level and whether the gene is identical to any of those identified on the other Atlantic salmon chromosome. S2B) Identified putatively functional Atlantic salmon β hemoglobin genes with chromosome, sequence contig number and approximate location (kb), strand of transcription, most highly similar Atlantic salmon EST cluster (if any), whether the gene has a corresponding full-length EST, whether the gene matches any of the previously identified Atlantic salmon hemoglobin genes at the amino acid level, whether the gene is identical to any of those identified on the other Atlantic salmon chromosome, and whether the β hemoglobin gene possesses the hallmarks of lacking the Bohr effect. S2C) Putatively identified Atlantic salmon hemoglobin pseudogenes with chromosome, sequence contig, location (kb), direction and descriptions of each exon. Click here for file

Additional file 3 **Figure S1: Dot plot comparing the sequenced BACs from Atlantic salmon chromosomes 3 and 6.** Regions surrounding the hemoglobin genes are > 95% similar. The dot plot was generated using the software JDotter [39]. The shared non-hemoglobin genes [*Dedicator of cytokinesis 6 *(*DOCK6*), *Dedicator of cytokinesis 7 *(*DOCK7*) and *Rhomboid 5 homolog 1*] within these regions are indicated. For chromosome 3, seven parts (P1-P7) are shown (bottom axis), representing seven sequence contigs, the first of which (sequence contig 49) does not contain any hemoglobin genes and is therefore not shown in Figure 1. Click here for file

Additional file 4 **Table S3: Putative α and β hemoglobin genes from other teleosts and *Xenopus tropicalis *used to generate phylogenetic trees.** The table lists all predicted intact α and β hemoglobin genes indentified within Biomart [69] for teleost genomes that have been sequenced and annotated (medaka, zebrafish, tetraodon, danio) and *Xenopus tropicalis*, which was used as an outgroup. For each hemoglobin gene identified, the table lists the species, chromosome or scaffold, start and stop positions, strand of transcription, Ensembl gene ID and our assigned gene name used in the phylogenetic trees. Click here for file
