# Supplementary material for: Genomic organization and evolution of the Atlantic salmon hemoglobin repertoire
Source: BMC Genomics. 2010 Oct 5;11:539. doi: 10.1186/1471-2164-11-539 (PMC3091688; doi:10.1186/1471-2164-11-539)
Supplement: Additional file 1 — Table S1: Primer and probe sequences. a ~40-mer forward primers were also used as hybridization probes. [file 1471-2164-11-539-S1.PDF]

Table S1: Primer and probe sequences

Primer name	Designed from	Forward	Reverse	Hybridization probe
S.salar alpha globin	Genbank X97289	TCTGGGGCAAGATTAAAGGA	GCTGCAAGGAATTTATCCACA	CACCCATGATGACGGCTCCATGCTTCTTGACTGGGGCAGA
S.salar beta globin	Genbank X97289	GCTGAATGCATCACCGTGT	ACAACGACAGCCAGGAACTT	CTGAATATCAGCACTGAAAACGGCGGGACCGAGCTTGGCG
S.salar non-Bohr beta globin	Genbank X97289	ACGCTGAGAAGAGCACCATC	GGCTGCGACAACCACTTT	CATCTCCGAAAGAGCCGAAATAACGCTGAGTCCAGGGGTA
O. mykiss embryonic alpha	Genbank AB015448	AGTTGAAGCAAAAATGAGTCTCTCCGCTAAGGACAAGGC	TGTCTTGGTCTGGGGGTACA	
O. mykiss embryonic beta globin	Genbank AB015450	GCAACATGGTTCAGTGGACAGACTTTGAGCGCGCCAC	GGGTCCAGGGGTACACAAC	
Ssa10067BSFU	S0055H05	ACAGCCTCAGACACCATTAC	ACACACGATCAACCCATATT	
Ssa10051BSFU	S0155C07	CAACCCTTATCCCTCTACCT	CAACCCTTATCCCTCTACCT	

[a] ~40-mer forward primers were also used as hybridization probes.
