# Supplementary material for: Genomic organization and evolution of the Atlantic salmon hemoglobin repertoire
Source: BMC Genomics. 2010 Oct 5;11:539. doi: 10.1186/1471-2164-11-539 (PMC3091688; doi:10.1186/1471-2164-11-539)
Supplement: Additional file 2 — Table S2A-C: Identified Atlantic salmon putatively functional and pseudogenized hemoglobin genes. S2A) Identified putatively functional Atlantic salmon α hemoglobin genes with chromosome, sequence contig number and approximate location (kb), strand of transcription, most highly similar Atlantic salmon EST cluster (if any), whether the gene has a corresponding full-length EST, whether the gene matches any of the previously published Atlantic salmon hemoglobin genes at the amino acid level and whether the gene is identical to any of those identified on the other Atlantic salmon chromosome. S2B) Identified putatively functional Atlantic salmon β hemoglobin genes with chromosome, sequence contig number and approximate location (kb), strand of transcription, most highly similar Atlantic salmon EST cluster (if any), whether the gene has a corresponding full-length EST, whether the gene matches any of the previously identified Atlantic salmon hemoglobin genes at the amino acid level, whether the gene is identical to any of those identified on the other Atlantic salmon chromosome, and whether the β hemoglobin gene possesses the hallmarks of lacking the Bohr effect. S2C) Putatively identified Atlantic salmon hemoglobin pseudogenes with chromosome, sequence contig, location (kb), direction and descriptions of each exon. [file 1471-2164-11-539-S2.PDF]

Table S2A: *S. salar* putatively functional alpha globin genes

Assigned name	Chromosome	Sequence contig	~Location (kb)	Strand	Matching *S.salar* fl-cDNA	Top globin EST cluster, %ID	Identical with: published globin	Identical with: other *S.salar* globin
SsaChr6α1	6	41	55	neg	BT067549.1	none	none	none
SsaChr6α2	6	41	66	pos	none	none	none	none
SsaChr6α3	6	41	79	neg	BT060399.1	Contig14853, 93.5%	none	none
SsaChr6α4	6	41	85	neg	BT060167.1	none	X97288, X97289	none
SsaChr6α5	6	41	124	neg	BT050340.1	none	X97285	SsaChr3α2$_1$ & SsaChr3α2$_2$
SsaChr6α6	6	41	130	neg	BT049299.1	none	X97286 (M-L @aa32)	none
SsaChr3α1	3	50	52	neg	BT047549.1	CLUSTER_ID#3953556, 95.8%	none	none
SsaChr3α2	3	58	66	pos	none	none	none	none
SsaChr3α3	3	47	119	neg	BT050355.1	none	X97284, X97285	SsaChr6α5
SsaChr3α4	3	47	124	neg	BT060387.1	none	none	none
SsaChr3α5	3	55	152	neg	BT060387.1	none	none	none
SsaChr3α6	3	55	172	neg	BT050355.1	none	X97284, X97285	SsaChr6α5
SsaChr3α5	3	56	180	neg	BT059945.1	none	none	none

Table S2B: *S. salar* putatively functional beta globin genes

Assigned name	Chromosome	Sequence contig	~Location (kb)	Strand	Matching *S.salar* fl-cDNA	Top globin EST cluster; %ID	Identical with: published globin	Identical with: other *S.salar* globin	Bohr status
SsaChr6ß1	6	41	64	pos	none	none	none	none	Bohr
SsaChr6ß2	6	41	71	neg	none	none	none	none	Bohr
SsaChr6ß3	6	41	82	pos	none	none	X97288, X97289 (V-L@ aa143)	none	Bohr
SsaChr6ß4	6	41	87	pos	BT048854.1	Contig36874, 98.5%	X97287	SSaChr3ß4	non-Bohr
SsaChr6ß5	6	41	127	pos	BT048961.1	none	X97285, X97286	none	Bohr
SsaChr6ß6	6	41	133	pos	none	none	X97289 (non-Bohr)	none	non-Bohr
SsaChr3ß1	3	58	84	neg	none	none	none	none	Bohr
SsaChr3ß2	3	58	90	neg	none	none	none	none	Bohr
SsaChr3ß3	3	46	111	pos	BT048802.1	Contig35020	none	none	non-Bohr
SsaChr3ß4	3	47	121	pos	BT060157.1	none	none	none	Bohr
SsaChr3ß5	3	47	127	pos	BT060187.1	Contig35020, 96.4%	none	none	non-Bohr
SsaChr3ß6	3	55	155	pos	BT050121.1	Contig35020, 97.3%	X97289 (non-Bohr)	SSaChr6ß4	non-Bohr
SsaChr3ß7	3	55	175	pos	BT056839.1	Contig38667, 95.5%	X97284	none	Bohr
SsaChr3ß8	3	56	182	pos	none	Contig35020, 96.9%	none	none	non-Bohr

Table S2C: *S. salar* putative globin pseudogenes

Assigned name	Chromosome	Sequence contig	~Location (kb)	Strand	Exon 1	Exon 2	Exon 3
SsaChr6ßψ1	6	41	48	neg	missing	frame shift	missing
SsaChr6αψ1	6	41	50	pos	good	truncated by insertion of stop codon	frame shift due to deletion
SsaChr6ßψ2	6	41	60	neg	good	insertion of stop codon mid exon	loss of stop codon
SsaChr6αψ2	6	41	61	pos	good	good	frame shift
SsaChr6ßψ3	6	41	68	neg	good	insertion of stop codon mid-exon	good
SsaChr3ßψ1	3	50	58	neg	missing	good	good
SsaChr3ßψ2	3	58	79	pos	missing	good	good
SsaChr3αψ1	3	58	61	neg	good	frame shift; deletion of 42 bp mid-exon	missing 33 bp at beginning of exon
SsaChr3ßψ3	3	47	133	pos	missing	good	truncated by insertion of stop codon
