# Supplementary figures and images for: Genomic organization and evolution of the Atlantic salmon hemoglobin repertoire
Source: BMC Genomics. 2010 Oct 5;11:539. doi: 10.1186/1471-2164-11-539 (PMC3091688; doi:10.1186/1471-2164-11-539)

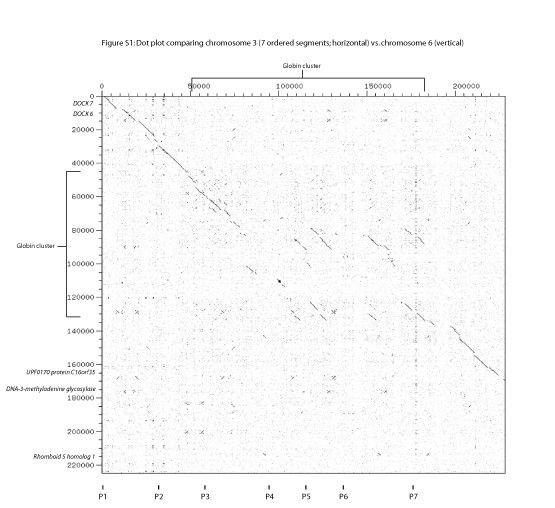

Supplement: Additional file 3 — Figure S1: Dot plot comparing the sequenced BACs from Atlantic salmon chromosomes 3 and 6. Regions surrounding the hemoglobin genes are > 95% similar. The dot plot was generated using the software JDotter [39]. The shared non-hemoglobin genes [Dedicator of cytokinesis 6 (DOCK6), Dedicator of cytokinesis 7 (DOCK7) and Rhomboid 5 homolog 1] within these regions are indicated. For chromosome 3, seven parts (P1-P7) are shown (bottom axis), representing seven sequence contigs, the first of which (sequence contig 49) does not contain any hemoglobin genes and is therefore not shown in Figure 1. [file 1471-2164-11-539-S3.JPEG]
