# Supplementary material for: Genomic organization and evolution of the Atlantic salmon hemoglobin repertoire
Source: BMC Genomics. 2010 Oct 5;11:539. doi: 10.1186/1471-2164-11-539 (PMC3091688; doi:10.1186/1471-2164-11-539)
Supplement: Additional file 4 — Table S3: Putative α and β hemoglobin genes from other teleosts and Xenopus tropicalis used to generate phylogenetic trees. The table lists all predicted intact α and β hemoglobin genes indentified within Biomart [69] for teleost genomes that have been sequenced and annotated (medaka, zebrafish, tetraodon, danio) and Xenopus tropicalis, which was used as an outgroup. For each hemoglobin gene identified, the table lists the species, chromosome or scaffold, start and stop positions, strand of transcription, Ensembl gene ID and our assigned gene name used in the phylogenetic trees. [file 1471-2164-11-539-S4.PDF]

Table S4: Putative α and β globin genes from teleosts and *Xenopus tropicalis* used to generate phylogenetic trees

Species	Ensembl gene ID	Assigned globin name	Chromosome/Linkage group	Start position	Stop Position	Strand
Gasterosteus aculeatus	ENSGACG00000013869	GacGroupXIα1	groupXI	13640461	13641149	pos
Gasterosteus aculeatus	ENSGACG00000013871	GacGroupXIβ1	groupXI	13642596	13643293	neg
Gasterosteus aculeatus	ENSGACG00000013873	GacGroupXIα2	groupXI	13644467	13645155	pos
Gasterosteus aculeatus	ENSGACG00000013876	GacGroupXIβ2	groupXI	13646453	13647149	neg
Gasterosteus aculeatus	ENSGACG00000013880	GacGroupXIα3	groupXI	13649883	13650298	pos
Gasterosteus aculeatus	ENSGACG00000013882	GacGroupXIβ3	groupXI	13651607	13652304	neg
Gasterosteus aculeatus	ENSGACG00000013889	GacGroupXIα4	groupXI	13653435	13654112	pos
Gasterosteus aculeatus	ENSGACG00000013891	GacGroupXIβ4	groupXI	13655329	13656035	neg
Gasterosteus aculeatus	ENSGACG00000013895	GacGroupXIβ5	groupXI	13659406	13660115	neg
Gasterosteus aculeatus	ENSGACG00000013902	GacGroupXIα5	groupXI	13661206	13661964	pos
Gasterosteus aculeatus	ENSGACG00000013918	GacGroupXIβ6	groupXI	13662623	13663375	neg
Danio rerio	ENSDARG00000069734	DreChr3β1	3	53994984	53995703	neg
Danio rerio	ENSDARG00000069735	DreChr3α1	3	53997339	53998149	pos
Danio rerio	ENSDARG00000077603	DreChr3β2	3	54002211	54003026	neg
Danio rerio	ENSDARG00000079078	DreChr3α2	3	54003866	54009713	pos
Danio rerio	ENSDARG00000078255	DreChr3β3	3	54007199	54008014	neg
Danio rerio	ENSDARG00000069747	DreChr3β4	3	54017851	54035806	pos
Danio rerio	ENSDARG00000077925	DreChr3α3	3	54024137	54024820	neg
Danio rerio	ENSDARG00000075934	DreChr3α4	3	54031030	54042878	neg
Danio rerio	ENSDARG00000079305	DreChr3α5	3	54050356	54051485	neg
Danio rerio	ENSDARG00000045142	DreChr12α1	12	19434748	19435868	neg
Danio rerio	ENSDARG00000045143	DreChr12β1	12	19437688	19438419	pos
Danio rerio	ENSDARG00000038147	DreChr12β2	12	19447742	19448842	pos
Danio rerio	ENSDARG00000045144	DreChr12α2	12	19451012	19452198	neg
Tetraodon nigroviridis	ENSTNIG00000018576	TniChr3α1	3	12162065	12163192	pos
Tetraodon nigroviridis	ENSTNIG00000018577	TniChr3α2	3	12165258	12166008	pos
Tetraodon nigroviridis	ENSTNIG00000012914	TniChr2α1	2	5887493	5888390	neg
Tetraodon nigroviridis	ENSTNIG00000012912	TniChr2α2	2	5891745	5893323	neg
Oryzias latipes	ENSORLG00000005210	OlaChr8α1	8	8382315	8408350	neg
Oryzias latipes	ENSORLG00000005230	OlaChr8β1	8	8384089	8409968	pos
Oryzias latipes	ENSORLG00000005248	OlaChr8β2	8	8388874	8389479	neg
Oryzias latipes	ENSORLG00000005267	OlaChr8α2	8	8390431	8391008	pos
Oryzias latipes	ENSORLG00000005283	OlaChr8β3	8	8404752	8405453	neg
Oryzias latipes	ENSORLG00000005297	OlaChr8α3	8	8411256	8412025	neg
Oryzias latipes	ENSORLG00000002990	OlaChr19α1	19	1478030	1480828	pos
Oryzias latipes	ENSORLG00000003020	OlaChr19β1	19	1484415	1562342	neg
Oryzias latipes	ENSORLG00000003046	OlaChr19α2	19	1486753	1487847	pos
Xenopus tropicalis	ENSXETG00000024628	XtrScaffold_733β1	scaffold_733	222200	223401	pos
Xenopus tropicalis	ENSXETG00000011556	XtrScaffold_733β2	scaffold_733	295422	296469	pos
Xenopus tropicalis	ENSXETG00000024627	XtrScaffold_733β3	scaffold_733	308259	309374	pos
Xenopus tropicalis	ENSXETG00000024626	XtrScaffold_733β4	scaffold_733	334930	336634	pos
Xenopus tropicalis	ENSXETG00000025667	XtrScaffold_357β1	scaffold_357	931132	932667	neg
Xenopus tropicalis	ENSXETG00000025664	XtrScaffold_357α1	scaffold_357	939457	940502	neg
Xenopus tropicalis	ENSXETG00000025663	XtrScaffold_357α2	scaffold_357	952535	956311	neg
Xenopus tropicalis	ENSXETG00000001141	XtrScaffold_357α3	scaffold_357	973850	975539	neg
Xenopus tropicalis	ENSXETG00000025662	XtrScaffold_357α4	scaffold_357	1003882	1005964	neg
Xenopus tropicalis	ENSXETG00000025661	XtrScaffold_357α5	scaffold_357	1015409	1016929	neg
Xenopus tropicalis	ENSXETG00000001139	XtrScaffold_357α6	scaffold_357	1031753	1032272	neg
Xenopus tropicalis	ENSXETG00000001137	XtrScaffold_357α7	scaffold_357	1133361	1134743	neg
Oncorhynchus mykiss	GenBank: AB015448	Omyα2emb	Unknown	Unknown	Unknown	Unknown
Oncorhynchus mykiss	GenBank: AB105449	Omyα1emb	Unknown	Unknown	Unknown	Unknown
Oncorhynchus mykiss	GenBank: AB015450	Omyβ1emb	Unknown	Unknown	Unknown	Unknown
Oncorhynchus mykiss	GenBank: AB015451	Omyβ2emb	Unknown	Unknown	Unknown	Unknown
